# Recurrent abdominal wall endometriosis at the trocar site of laparoscopy: A rare case

**Published:** 2018-10

**Authors:** Mojgan Akbarzadeh-Jahromi, Maedeh Motavas, Afsoon Fazelzadeh

**Affiliations:** 1 *Maternal-Fetal Medicine Research Center, Pathology Department, Shiraz University of Medical Sciences, Shiraz, Iran.*; 2 *Pathology Department, Shiraz University of Medical Sciences, Shiraz, Iran.*; 3 *Shiraz University of Medical Sciences, Shiraz, Iran.*

**Keywords:** Endometriosis, Laparoscopy, Abdominal wall, Hernia

## Abstract

**Background:**

Surgical scar endometriosis is typically presented as a slow-growing, painful abdominal mass near the site of a past surgery. Endometriosis on the trocar port site is rare. To best of our knowledge, only 17 cases have been reported in the literature. The nonspecific nature of endometriosis presents a diagnostic challenge, and it is often considered as an incisional hernia or other conditions.

**Case:**

Here, we described our experience with a recurrent abdominal scar endometriosis case at the trocar port site of a previous laparoscopy, which was initially thought to be an incisional hernia.

**Conclusion:**

Abdominal wall endometriosis should be considered as an important differential diagnosis in females with a positive history of prior abdominal surgery, presented with painful nodule or mass at the site of the surgery.

## Introduction

Endometriosis is defined as the presence of functional endometrial glands and stroma in extrauterine locations. The endometrial tissue is usually found within the ovaries, uterine ligaments, fallopian tube, or pelvic side wall (1, 2). Extra-pelvic sites have been reported in abdominal wall, extremities, lungs, and brain. The most common symptoms of endometriosis include dysmenorrhea, chronic pelvic pain, and infertility (1, 2). Abdominal endometriosis is an uncommon condition. It is caused by the dissemination of endometrial tissue into the wound at the time of abdominopelvic surgeries, and interventions such as hysterotomy, salpingostomy, episiotomy, cesarean section, appendectomy, amniocentesis, and laparoscopy (2). Endometriosis on the trocar port site is rare. To best of our knowledge, only 17 cases have been reported in the literature, but no recurrent case has been reported (3). Here, we have described a case of recurrent endometriosis at the site of previous abdominal laparoscopy, which was initially diagnosed as an incisional hernia.

## Case report

A 25-yr-old G1L1 women was presented with abdominal pain and swelling at the port site of the previous laparoscopy. The patient was a case of primary infertility and polycystic ovary syndrome. She had received many medications such as clomiphene, human menopausal gonadotropin/ human chorionic gonadotropin, metformin, and levothyroxine without any response. All of her husband’s work ups were normal. Hence, she underwent diagnostic laparoscopy, and both ovarian cauterizations were performed due to polycystic ovary syndrome, seven yr ago. 

She became pregnant after two months, and with normal vaginal delivery, a boy was born. After one-yr mild abdominal pain began and worsened during the upcoming years. So, she was referred to a general surgeon with the impression of incisional abdominal wall hernia, therefore, herniorrhaphy and mesh repair was done for her. However, her abdominal pain continued after surgery with the cyclic pattern, and after seven months a large mass palpated at the site of abdominal scar. In a clinical examination, a painful, round, firm mass was detected near the previous site of operation. The patient underwent a second operation, and an irregular fibro-fatty tissue measuring 4x4x1.5 cm was excised. 

**Figure 1:**
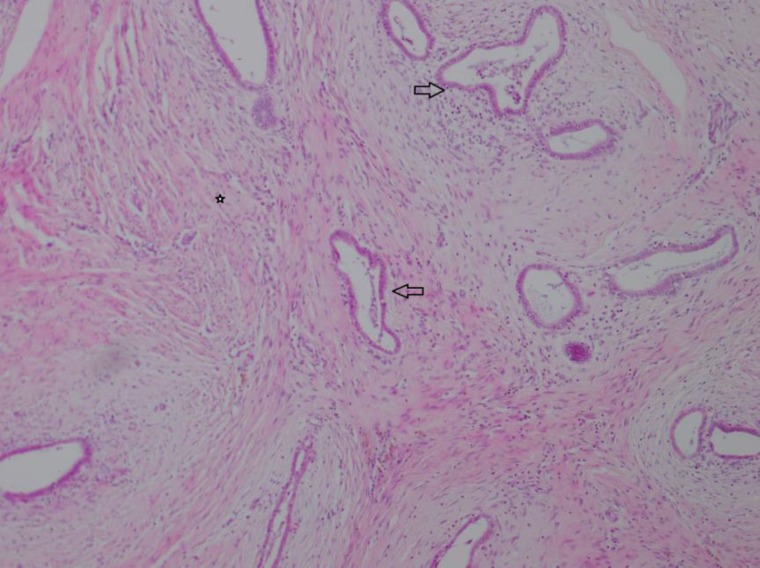
Endometrial stroma and glands (arrow) in abundant desmoplastic tissue (asterisks) in abdominal wall .x40 H & E

**Figure 2:**
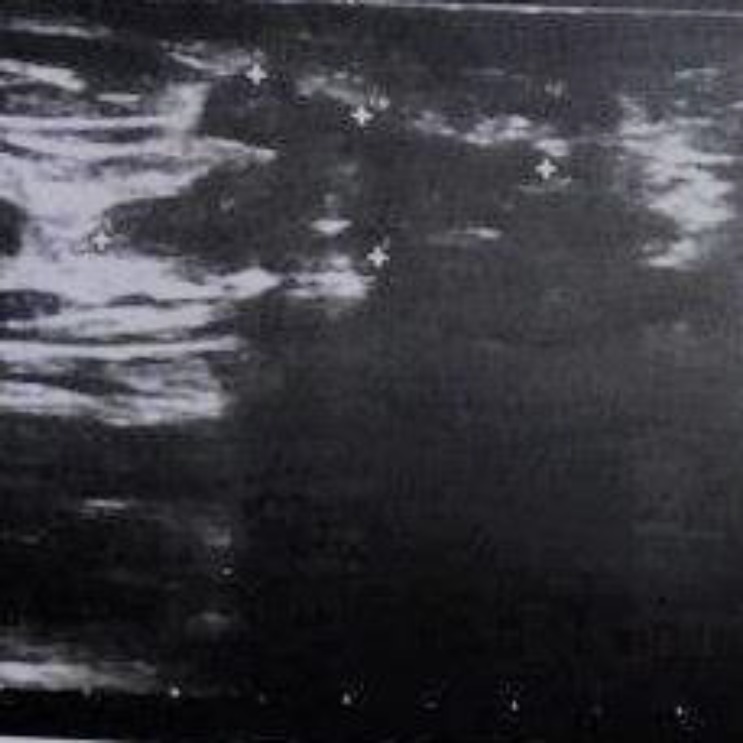
Ultrasonography image shows a 26x12x25mm vascularized, hypoechoic, irregular in shape lesion in the abdominal wall, 7mm deep to the skin

Serial cut sections of the specimen showed yellow-brown color with some foci of hemorrhage. Microscopic examinations showed endometrial glands and stroma in favor of endometriosis (Figure 1). After the second operation, the patient also had a recurrence of endometriosis in ultrasonography (Figure 2) that relatively has good response to medical therapy and now the patient is pregnant (G2). No evidence of ovarian endometriosis was observed in the primary laparoscopic examination, and also in the follow-up ultrasonography after abdominal wall surgery. 

**Ethical consideration**

Written informed consent was obtained from the patient for the publication of this case report as well as accompanying images. A copy of the written consent is available for review by the Editor-in-Chief of this journal.

## Discussion

Abdominal wall endometriosis (AWE) is characterized by the presence of a nodule along the surgical scar. There is history of previous surgery such as C-sections, hernias, laparotomies, laparoscopy port sites or hysterotomies when the endometriosis involves the abdominal wall (2-6). Frequent risk factor for AWE are elevated body mass index, multiparous women and surgeries of uterine cavity. The most common surgery that is associated with AWE is the C-section (4, 6). The patient may present the symptoms several years after the surgical procedure with an average of 7 yr (range 1-32 yr) (4). The incidence of abdominal wall endometriosis varies from 0.03-0.8% (6). 

Several theories for endometrioma formation has been proposed, which include the hematogenous, lymphatic and iatrogenic spread of endometrial cells, metaplastic change of pluripotent cells at any site to endometrial cells, the retrograde spread of endometrial cells to the pelvis during menstruation, and immune system dysfunction (3). Scar endometrioses are considered to be the result of direct inoculation of endometrial cells into the abdominal fascia or subcutaneous tissue during surgical intervention which subsequently stimulated by estrogen to produce endometrioses (2, 3). This theory is showed in studies, in which normal menstrual outflow inoculated to the abdominal wall induce subcutaneous endometriosis (7). 

The diagnosis of abdominal wall endometriosis is mostly clinical when there is a mass at the surgical scar site, which becomes painful during menstruation (2, 6). However, cases with asymptomatic palpable mass with noncyclical pain and cases with a delay time between surgery and onset of symptom can bear a suspicion to various conditions. Therefore, the preoperative diagnosis in these patients varies from an incisional hernia, as in our case, to metastatic cancer (2, 7). Although the sensitivity of radiologic modalities is high, they are not able to distinguish amongst several types of masses of the abdominal wall. Computerized tomography findings may help to diagnose, exclude, or suggest the presence of a mass and define its extent and nature. Magnetic resonance imaging provides a better contrast resolution than computerized tomography or ultrasound sonography (8). 

Fine-needle aspiration cytology has been shown to be a fast, accurate, and inexpensive method to make a diagnosis before surgery. The most specific findings are endometrial-like epithelial cells, stromal cells, and hemosiderin-laden macrophages (9). Several treatment options have been suggested including pharmacological or surgical treatment, but the definitive treatment of abdominal wall endometriosis and the gold standard is wide surgical excision with 5-10 mm negative margins to prevent recurrence (10).

In our case, the presence of endometriosis and its sign and symptoms were 7 yr after the diagnostic laparoscopy at the trocar port site that was first operated as an incisional hernia, and it recurred twice. At first, it reoccurred seven months later and once again after second surgery. To best of our knowledge, there are only a few cases of recurrent scar endometriosis after excision (3). In a retrospective review by Kang and colleagues of 37 abdominal wall endometriosis cases; there was only one recurrence after 34 months from pervious surgery (5). 

However, the patient received gonadotropin-releasing hormone-agonist as treatment. Gonzalez-Fernandez presented a case report of a 44 yr old woman with recurrent abdominal wall endometriosis, noted on the fascia, which was resected 9 yr prior to its recurrence (6). The risk factors for recurrence are the size of mass and depth of infiltration, especially when peritoneal and /or abdominal wall muscle involvement is present. Pelvic endometriosis and scar endometriosis co-occurrence have been reported in about 14.3-26% of cases (2). Therefore, patients should be examined for synchronous pelvic endometriosis. Postoperative follow up by a gynecologist is recommended (2).

## Conclusion

Abdominal wall endometriosis should be considered as an important differential diagnosis in females with a positive history of prior abdominal surgery, presented with painful nodule or mass at the site of the surgery.
